# Orchitis

**Published:** 1884-03-20

**Authors:** H. M. Lawson

**Affiliations:** Georgia


### ORCHITIS.

BY H. M. LAWSON, M.D., OF GEORGIA.

A recent case or two of orchitis brought to mind a little contrivance of my younger days, when I more often had to prescribe for this difficulty. In those days I tried the bandage, adhesive straps and suspensories of different kinds; but, as they all failed to meet the indications as perfectly as I thought they ought, I endeavored to do so by trying plans of my own. After various contrivances, more novel than useful, I hit upon the following, which answered the purpose admirably:

Take a piece of cheap, thin homespun, about three and a half inches long and three-fourths of an inch wide, double it the long way and sew the edges together; then fill with cotton, not too tightly, sew a button on one end and make a button-hole in the other; this, when buttoned, will form a ring. All that is necessary in addition to this is a piece of the same kind of homespun four inches square. Apply as follows: Put the center of the square piece of cloth to the center of the swollen testicle below, bring the edges and corners up around, completely inclosing it, then button the ring around above the testicle, which will hold it in position. Now draw upon the edges and corners of the square piece all around until sufficient compression is made. The sound testicle is to be left above the ring.

If too much compression is made it is easily removed by drawing the cloth in the opposite direction: it is easily increased or diminished at will. Its other advantage will be apparent on using it. Easily obtained, applied and adjusted, I feel sure that no one who tries this method will afterward use any other.

I think I have seen it somewhere, lately, advised to omit compression altogether, and I think, too, that this was due to the difficulty of satisfactorily applying compression.

